# Global Public Interest in CKD Based on Internet Searches

**DOI:** 10.3389/fmed.2021.564967

**Published:** 2021-01-22

**Authors:** Yang Liu, Xu-jie Zhou

**Affiliations:** Key Laboratory of Renal Disease, Ministry of Health of China, Key Laboratory of Chronic Kidney Disease Prevention and Treatment (Peking University), Ministry of Education, Renal Division, Peking University First Hospital, Peking University Institute of Nephrology, Beijing, China

**Keywords:** chronic kidney disease, internet, search, nephritis, Google trends

## Introduction

Kidney disease has a major effect on global health as a direct cause of global morbidity. Chronic kidney disease (CKD) is largely preventable and treatable. Thus, it deserves greater attention in global health policy decision making (1). The increasing economic and social burdens have spurred additional research interest in epidemiology, geographic prevalence, and cost-effectiveness strategies in intervention and prevention. Global public interest in seeking information about CKD may provide important clues, such as screening and awareness. In recent years, internet data have integrated into health informatics, and has been recognized as a valuable tool for estimating health-related needs. By generating big data according to specified keywords, Google trends (GT) is one of the most popular and efficient way in examining human behavior (2, 3). To our knowledge there have been no studies that examined whether search queries in GT search volume could direct educational campaigns, monitor and address trends in real-time for CKD. To explore internet search trends data as a unique resource for monitoring online health information-seeking behavior, this study aims to investigate the global public interests in seeking information of CKD using GT.

## Methods

Google trends were queried Search (http://google.com/trends) using terminology related to CKD in English from 2004 through 2019. The resulting Comma-Separated Values (CSV) files were exported. The search volumes were not given the actual volume but in what Google terms a “search volume index” (SVI) from 0 to 100, representing the relative search volume for a search term indexed against the overall search volume. Peak search activity over a given period was graded as 100% and activity at all other time periods was presented relative to that peak. As some causes of CKD are rarely searched, we restricted terms including “nephritis,” “IgA nephropathy,” and “lupus nephritis” to check their etiology trends. The query was also searched within different countries to summarize regional interest or economic status. We adjusted influence of different days (i.e., adj RSV of CKD = relative search volume (RSV)/total days of the searching month × 365.25/12) and also adjusted month item in seasonal analysis, as previously reported (4). Statistical analysis was performed using the computing environment R-3.6.2 and GraphPad Prism 8.

## Results

**Figure 1:**
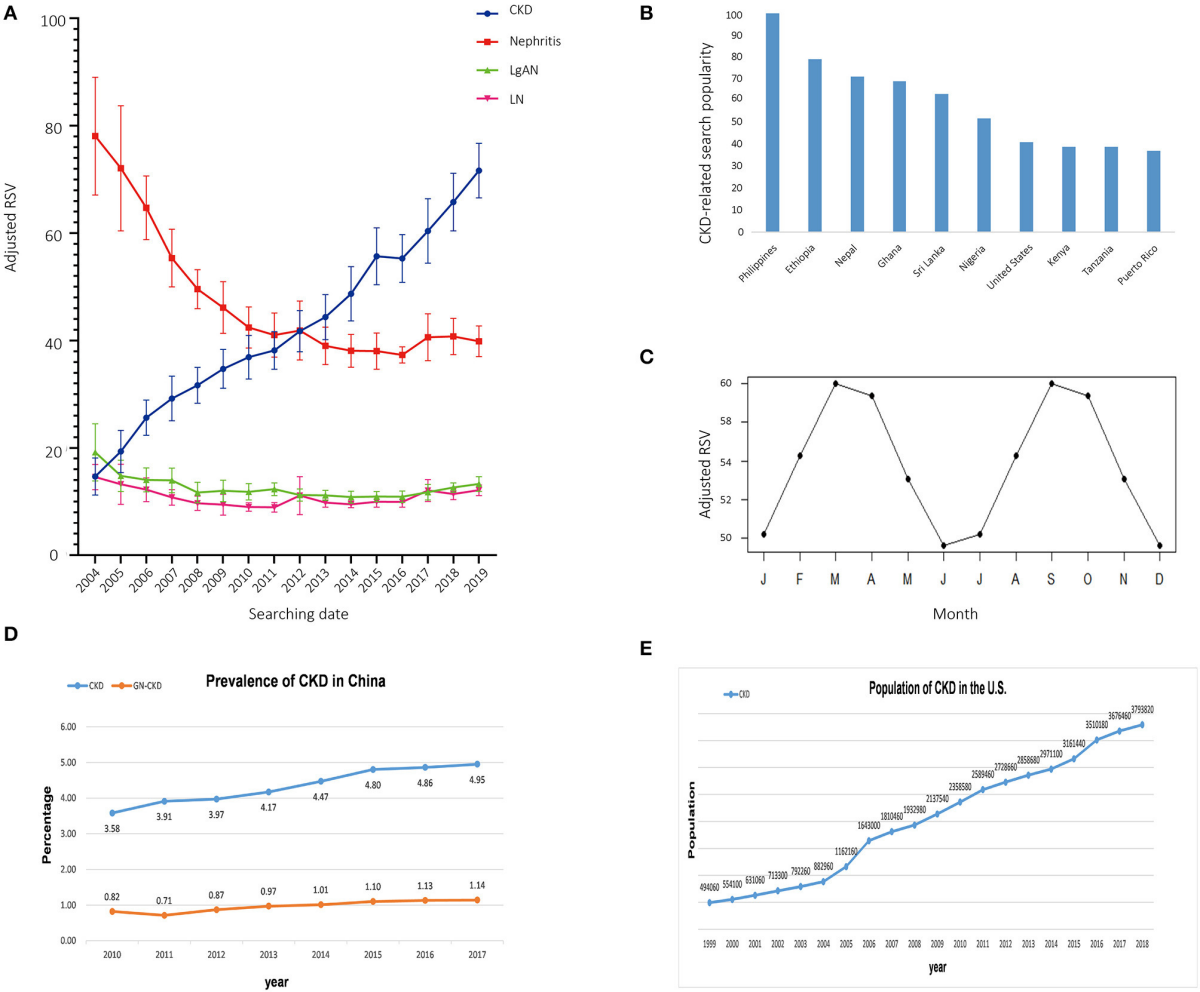
Google trends in CKD. **(A)** The trend for adjusted relative searching volume (RSV) on CKD, nephritis, IgA nephropathy, and lupus nephritis. Data were presented as mean ± *SD*. **(B)** The top 10 countries with the highest RSV on searching item of “chronic kidney disease.” **(C)** Cosinor models for seasonal variation in the RSV of CKD. **(D)** Trends in chronic kidney disease (CKD) and CKD due to glomerulonephritis (GN-CKD) among hospitalized patients in China. **(E)** Trends in prevalence of recognized CKD in the US by USRDS.

As can be seen from Figure 1A, global searches for CKD were gradually increasing. With regard to etiology, it indicated a declining trend on the RSV for nephritis. In 2004, the RSV for nephritis was 4–5 times as that of CKD in the same period. In 2019, nephritis search volume decreased to 1/3 of 2004, which was 1/2 of RSV for CKD in the same period. Further analysis of the most common forms of primary (IgA nephropathy) and secondary nephritis (lupus nephritis), it can be observed that they showed a downward trend first, then remained stable and low in the past 10 years. Most of the top 10 regions with the highest RSV in searching CKD were developing countries (Figure 1B). With regard to seasonal variation, it can be observed in Figure 1C, months from March to April and September to October were higher than other months (Cosinor test, *p* = 1.78 × 10^−6^).

To correlate RSV with the actual incidence of CKD, we retrieved epidemiology data for CKD in China and the United States, representing developing county and highly developed country. The respective data were from publication of China Kidney Disease Network (CK-NET) 2016 Annual Data Report and the 30th Annual Data Report (ADR) of the United States Renal Data System (USRDS) (5–7). Consistent with the public interest trend shown in GT, actual epidemiological data in China and US showed an increase in CKD prevalence (Figures 1D,E).

## Discussion

The increasing trend of public search for CKD, suggested a global, and regional burden of CKD. The data from Internet sources may serve as a real-time surveillance tool and an alert for healthcare systems, so as to allocate appropriate resources for global health policy decision making (7). It may be particularly important in locations with low and middle incomes. Those locations may have limited capacity to establish national databases for certain specific disease. While web-based data reduce the cost of obtaining statistics, and could be regarded as a replacement for traditional surveillance networks. Google web search logs provide one of the most timely, broad-reaching disease monitoring systems available. Whereas, traditional systems may require several weeks or months to gather and process surveillance data. With other healthcare surveillance systems, the GT data are useful to spur further investigation and collection of direct measures of disease activity in the whole nation or district. In the field of nephrology, different etiologies of kidney diseases may indicate very different therapy protocols and prognosis. Our above data may also suggest some important epidemiological changes [i.e., the proportion of nephritis showed decreasing trend, which has been observed in fast-growing country like China (8)]. Environmental factors in CKD, such as temperature, or pollution, may account for the seasonal variation. GT can be recognized as an auxiliary tool for estimating epidemiology and gathering data about CKD patterns.

## Author Contributions

YL and X-jZ coordinated data collection, monitoring, and data analysis. All authors worked on the final version of this article.

## Conflict of Interest

The authors declare that the research was conducted in the absence of any commercial or financial relationships that could be construed as a potential conflict of interest.
